# Curcumin and Its Potential Impact on Microbiota

**DOI:** 10.3390/nu13062004

**Published:** 2021-06-10

**Authors:** Marzena Jabczyk, Justyna Nowak, Bartosz Hudzik, Barbara Zubelewicz-Szkodzińska

**Affiliations:** 1 Department of Nutrition-Related Disease Prevention, Faculty of Health Sciences in Bytom, Medical University of Silesia, 41-900 Bytom, Poland; marzena.jabczyk@gmail.com (M.J.); bzubelewicz-szkodzinska@sum.edu.pl (B.Z.-S.); 2 Department of Cardiovascular Disease Prevention, Faculty of Health Sciences in Bytom, Medical University of Silesia, 41-900 Bytom, Poland; bartekh@mp.pl; 3 Silesian Center for Heart Diseases, Third Department of Cardiology, Faculty of Medical Science in Zabrze, Medical University of Silesia, 41-800 Zabrze, Poland

**Keywords:** curcumin, antioxidant, microbiota, health

## Abstract

Curcumin is one of the most frequently researched herbal substances; however, it has been reported to have a poor bioavailability and fast metabolism, which has led to doubts about its effectiveness. Curcumin has antioxidant and anti-inflammatory effects, and has demonstrated favorable health effects. Nevertheless, well-reported in vivo pharmacological activities of curcumin are limited by its poor solubility, bioavailability, and pharmacokinetic profile. The bidirectional interactions between curcumin and gut microbiota play key roles in understanding the ambiguity between the bioavailability and biological activity of curcumin, including its wider health impact.

## 1. Introduction

Curcumin is a polyphenol substance isolated from the rhizome of Zingiberaceae and Araceae plants. It is a major active constituent of turmeric, a common Asian spice used as a dietary spice, food-coloring, as a herbal remedy, and in the beverage industries. Its bioactive components have been investigated recently [1,2]. Diferuloylmethane (1,7-bis(4-hydroxy-3-methoxyphenyl)-1,6-heptadiene-3,5-dione), which is commonly referred to as curcumin, has been shown to have activity at the cellular level, by signaling multiple molecules. In addition it exerts antioxidant and anti-inflammatory properties. It may have many therapeutic effects [2,3], having exhibited antitumor, chemosensitizing, hepatoprotective, lipid-modifying, and neuroprotective effects [4]. Nevertheless, well-reported in vivo pharmacological activities of curcumin are limited by its poor solubility, bioavailability, and pharmacokinetic profile. As many data have suggested, despite its low absorption, curcumin may demonstrate beneficial effects on health by influencing the intestinal barrier function, sustaining high concentrations in the intestinal mucosa, modulating the functioning of the intestinal barrier, and decreasing high concentrations of bacterial lipopolysaccharide (LPS) levels [5,6,7]. This review presents the mechanisms of action of curcumin and its potential influence for prevention and treatment by regulating the gut microbiota.

### 1.1. Curcumin Safety

The Joint United Nations and World Health Organization Expert Committee on Food Additives (JECFA) and the European Food Safety Authority (EFSA) have set the allowable daily intake (ADI) for curcumin at 0–3 mg/kg body weight. Some studies have reported negative side effects in healthy adults that received 500–12,000 mg in a dose response manner [2]. Some studies have showed that nausea, diarrhea, and elevated serum alkaline phosphatase and lactate dehydrogenase levels may be observed in subjects receiving 0.45–3.6 g/day [8]. On the other hand, Shabbir et al. [4] suggested that many studies have shown that it is safe to consume 8 g/day of curcumin.

### 1.2. Oral Bioavailability of Curcumin

Curcumin belongs to the family of polyphenol compounds, which has at least one aromatic ring structure with at least one hydroxyl group. Polyphenols are found in many vegetables and secondary metabolites, and derive from the shikimic acid pathway [9].

Factors impacting on oral curcumin bioavailability in the diet may be affected by food processing, macronutrients, and origin. In food, polyphenol composition is affected not only by heating changes but also drying, grinding, climate, and plant stress, among others. The poor availability of curcumin has resulted in the development of several curcumin formulations over the last decade, which could improve its bioavailability. Those formulations include curcumin nanoparticles, curcumin in lecithin, phosphatidylcholine carrier, and solid lipid curcumin nanoparticles [7].

As Shabbir et al. [4] emphasized, dietary lipids may affect the solubility and absorption of curcumin. The lipophilic nature of curcumin and the existence of hydroxyl groups in its structure allow it to be metabolized very rapidly in the kidneys and liver. More, it is unstable in most bodily fluids, such as water. Thus, it is recommended to be mixed with oil (or, even, milk) before consumption, in order to improve its absorption [4].

Moreover, Dei Cas and Ghidoni [10] pointed out that, to increase the dietary intake of curcuminoids, turmeric should be paired with lecithin-rich products, such as vegetable oils or eggs. In a recent study, Jardim et al. [11] demonstrated that the addition of lecithin is a promising strategy for improving the properties of nanoparticles such as curcumin, leading to its enhanced efficiency. 

Piperine is a natural alkaloid that is found in black pepper (*Piper nigrum*), which is capable of increasing the bioavailability of curcumin by inhibition of biotransformation—especially glucuronidation [10]—in the liver and small intestine [12]. As Hewlings and Kalman [13] emphasized in their work, piperine has been associated with an increase in the bioavailability of curcumin by 2000%. 

Interestingly, Schiborr et al. [14] revealed the existence of sex differences, with respect to the plasma levels of oral micellar curcumin. They have demonstrated that women absorbed curcumin to a larger extent than men (1.4-fold higher in women, in comparison to men). The researchers hypothesized that it may be explained by the increased expression and activity of drug efflux in the liver and some enzymes involved in curcumin biotransformation in men. It is worth mentioning that these results may also be due to differences in body weight, blood volume, and body fat. As such, this topic requires further research. 

The potential beneficial effects of curcumin depend on its dietary and supplementation intake, as well as the individual’s capacity for metabolism, which depends on the biodiversity of their microbiota.

Many studies have reported that high concentrations of curcumin after oral administration have been detected in the gastrointestinal tract. Scazzocchio et al. [15] noted the ambiguity between the low systemic bioavailability and broad pharmacological activity of curcumin. The hypothesis stated that polyphenol directly exerts regulatory effects on microbiota. In turn, Ng et al. [16], in their meta-analysis, suggested another thing which may contribute to the therapeutic effect of curcumin. They emphasized that poor oral bioavailability and the fact that curcumin ingested may be excreted in the feces unmetabolized lead to the point that, after digestion, curcumin reaches the intestine almost unchanged and that may demonstrate hypothetical beneficial effects on the intestinal microflora. Moreover, the potential beneficial effects depend largely on the dietary intake of curcumin, either in terms of an individual’s capacity for metabolizing it, or as a consequence of the composition of the intestinal microflora of individuals [15].

### 1.3. Curcumin Metabolism

The primary sites of metabolism for curcumin are the liver, together with the intestine and gut microbiota. After several reactions, curcumin’s double bonds are reduced in hepatocytes and enterocytes, and form dihydrocurcumin, tetrahydrocurcumin, hexahydrocurcumin, and octahydrocurcumin [10,17]. Following oral ingestion, the extensive metabolism of curcumin include reduction, sulfation, and glucuronidation in the liver, kidneys, and intestinal mucosa. 99% of plasma curcumin is present as glucuronide and sulphate conjugate metabolites which are less active [18]. Interestingly, a recent study has shown greater metabolic conjugation and reduction in the human gastrointestinal tract, in comparison to rat intestines [19]. 

Many other studies have revealed the presence of curcumin metabolites in the human intestinal tract [17]. Due to its resistance to low pH (i.e., stable in the range of pH 2.5 to 6.5), curcumin reaches the large intestine and undergoes extensive metabolic phases [15]. So far, two phases of curcumin metabolism have been reported [10,17,20]. 

Phase I includes the reduction of the four double bonds of the heptadiene-3,5-dione structure, reduction of curcumin to dihydrocurcumin (DHC), then to tetrahydrocurcumin (THC), and later to hexahydrocurcumin and, finally, octahydrocurcumin. 

In phase II which takes place in the intestinal and hepatic cytosol [10], curcumin and its reduced metabolites are conjugated with monoglucuronide, a monosulfate, and then a mixed sulfate/glucuronide (conjugated curcumin), followed by conjugated DHC, conjugated THC, conjugated hexahydrocurcumin and, finally, conjugated octahydrocurcumin [20]. Hexahydrocurcumin and THC are the major products observed in most studies, whereas DHC and octahydrocurcuminol are generally not detected at all [21]. The lack of these two products may be the result of the enzymes responsible for the bioreduction which have been found to reside in the cytosol of the liver and intestine. These compounds may be easily conjugated in vivo and in vitro. For instance, hexahydrocurcumin shares the same phenolic groups or diketo moieties as curcumin, but has no olefinic double bonds. This fact makes it more stable than curcumin at a physiological pH level of 7.4 [22].

**Figure 1:**
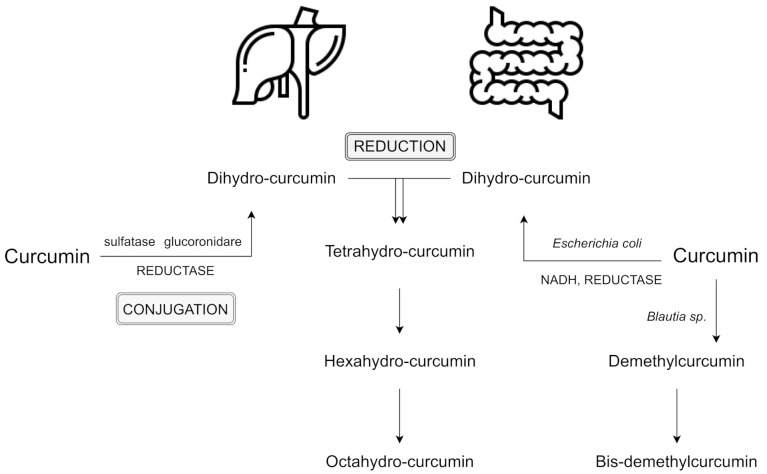
Reductive conjugative metabolism of curcumin and an alternative metabolism by intestinal microbiota; based on [10,20,24]. Abbreviation: NADH - nicotinamide adenine dinucleotide.

Interestingly, the intestinal microflora can deconjugate phase II metabolites and convert them back to the corresponding phase I metabolites, which may also lead to some fission products, such as ferulic acid [23]. Moreover, curcumin also may be metabolized alternatively by intestinal microflora, such as *Escherichia coli* and *Blautia* sp. *E. coli* has been reported to be active by an nicotinamide adenine dinucleotide phosphate (NADPH)-dependent reductase in a two-step reduction pathway from curcumin to DHC, then to THC. In turn, *Blautia* sp. carries out curcumin demethylation into two derivates: demethylcurcumin and bis-demethylcurcumin [10,24]. Curcumin and its reduced metabolites seem to be readily conjugated in vivo and in vitro [21]. Reductive conjugative metabolism of curcumin and an alternative metabolism by intestinal microbiota are shown in Figure 1.

Wang et al. [25] have recently developed a rapid LC-MS/MS ( consisting of an LC-20 AD parallel pump, a SIL-20A autosampler, a DGU-20A3R degasser unit, a CTO-20A column oven and an MS-8040 spectrometer ) method which evaluates the pharmacokinetics and tissue distribution of curcumin and its metabolites in mice. Fifty male mice were randomly divided into ten groups, and received 20 mg/kg curcumin. After drug administration, blood samples were drawn from the heart at various intervals. The study described decreased THC and DHC in plasma, while THC was present as a main metabolite of curcumin in plasma. Moreover, the results showed that curcumin and THC may be detected in the liver, and curcumin and DHC may be detected in the kidneys. Interestingly, only curcumin was detected in the brain, which (as suggested) means that curcumin may cross the blood-brain barrier [4,25].

In a recent article, Shabbir et al. [4] drew attention to these metabolites and their antioxidant, anti-inflammatory, and neuroprotective effects, considering the gut-brain axis and pathways such as the demethylation, reduction, acetylation, hydroxylation, and demethoxylation of curcumin. They highlighted the results of altered microbial abundance and biodiversity through curcumin biotransformation, exerting indirect health benefits in Alzheimer disease-induced in transgenic mice [26]. This topic needs further study.

### 1.4. Curcumin and Microbiota

The human intestinal microbiota contains micro-organisms, estimated to consist of more than 1000 bacterial species, which are indispensable for organism physiology and metabolism and, as a consequence, play key roles in maintaining general health [1]. To date, many studies have examined alterations in the composition of microbiota and its link to spectrum of diseases, including diabetes, obesity, inflammatory bowel, liver diseases, depression, psoriasis, and neurogenerative disorders, and the field is growing at a dynamic rate.

Interestingly, despite its poor plasma and tissue bioavailability, preferential distribution and accumulation of curcumin in the intestine has been reported after oral or intraperitoneal administration [1]. Several studies have examined whether curcumin may exert regulative effects on the microbiota community. Human intestinal microbiota can transform curcumin in various metabolism pathways, including producing active metabolites which are able to exert local and systemic effects, but also by reducing the heptadienone backbone and demethylation by *Blautia* spp. [5,24]. As Pandey et al. [17] have suggested, an alternative metabolism of curcumin occurs by the intestinal microflora, especially by *Escherichia coli*. Hassaninasab et al. [20] also identified micro-organisms (strain *Escherichia coli*) capable of converting curcumin, isolated from the feces of two healthy subjects. An NADPH-dependent enzyme (CurA) in *E. coli* converts curcumin into dihydrocurcumin (DHC) and tetrahydrocurcumin (THC) [17]. In turn, the results of a study on *Bifidobacteria* reported by Jazayeri et al. [27] presented that micro-organisms such as *Bifidobacteria pseudocatenulaum, Enterococcus faecalis, Bifidobacteria longum, Lactobacillus acidophilus,* and *Lactobacillus casei* are important bacterial strains which are capable of reducing the parent compound of curcumin more than 50% and, in this way, can metabolize curcumin.

Shen et al. [28] reported ananalysis of the intestinal microflora in curcumin-administered mice and controls by pyrosequencing the V3 and V4 regions of the bacterial 16S ribosomal RNA genes. The researchers investigated whether the concentration of curcumin in the intestines following oral administration may result in regulative effects on the intestinal microbiota. One group of mice (*n* = 6) received a standard diet enriched with natural mixtures isolated from turmeric, which contained the three major parts of curcumin (40.9%), demethoxycurcumin (33.2%), and bidemethoxycurcumin (23.3%), in a dose of 100 mg/kg body weight for 15 days. The control group (*n* = 6) was supplied with the same feed, but without curcumin gavage. Following the addition of curcumin, significant increases (*p* < 0.05) in several representative families in the gut, including *Prevotellaceae*, *Bacterioidaceae*, and *Rikenellaceae,* were recorded. The abundance of *Prevotellaceae* decreased significantly in the curcumin group, in comparison to controls (from 15.48% to 6.16%; *p* = 0.01). A significant reduction was also reported in *Prevotella* abundance (from 13.29% to 4.63%; *p* = 0.00), Conversely, *Bacteroidaceae* in the curcumin group was significantly higher in comparison to the control group (3.21% to 1.15%; *p* = 0.00). Similarly, the amount of *Rikenellaceae* increased (from 4.73% to 7.96%; *p* = 0.04) with *Alistipes* abundance (from 4.73% to 7.96%; *p* = 0.04). These results indicated that, despite no significant alteration, oral administration of curcumin tended to decrease the microbiome biodiversity. More studies are required to extend the current microbiome outcomes, in order to prove the therapeutic application of curcumin for the gut microbiome.

The action of curcumin (as well as resveratrol and simvastatin) has also been examined in animals affected by *Toxoplasma gondii*. It was found that curcumin, resveratrol, and simvastatin-treated animals had less proinflammatory *Enterobacteria* and *Enterococci* by 3.0–3.5 (*p* < 0.000001) and 1.0–1.5 (*p* < 0.0005), respectively. The potentially anti-inflammatory *Lactobacilli* or *Bifidobacteria* were slightly increased, by up to 1.0 log orders (*p* < 0.05–0.0005) [29].

**Figure 2:**
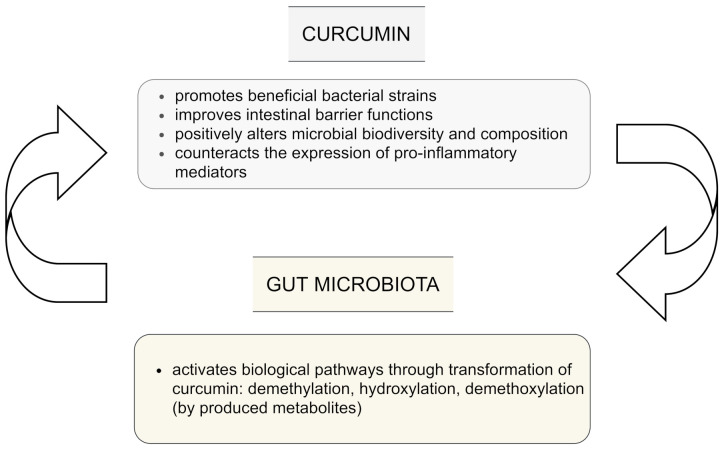
Interplay between curcumin and gut microbiota; based on [1,4,15,23].

The influence of curcumin on the intestinal microbiota is still not clear. However, current evidence indicates that curcumin is biotransformed not only in the gut to various metabolites via different pathways which include among others demethylation, hydroxylation, and demethoxylation. These metabolites have been reported to be even more bioactive than the parent curcumin, but also the gut microbiota may be regulated by administration of curcumin and lead to changes in the biodiversity and composition of microbes. This plays a key role in potential indirect health benefits [1]. The bi-directional interplay between curcumin and gut microflora is presented in Figure 2.

### 1.5. Curcumin and Microbiota in Liver Disease

Feng et al. [30] have investigated, using taxon-based analysis, the correlation between changes in gut microbiota regulation in curcumin-mediated attenuation and the development of non-alcoholic fatty liver disease in rats. Their data revealed that curcumin treatment significantly changed the gut microbial composition. The operational taxonomic units that were altered by curcumin treatment were related to hepatic steatosis parameters. Types such as *Spirochateae, Tenericutes*, and *Elusimicrobia* were decreased, conversely to *Actinobacteria*, which were markedly increased. In their discussion, they paid attention to the immunomodulatory effects of curcumin, due to its alteration in intestinal bacterial communities, which led to the relative abundance of short-chain fatty acid (SCFA) producing bacteria (including *Blautia* and *Allobaculum*). That may contribute to the alleviation of inflammation, insulin resistance, and obesity [31]. As a consequence, it may serve as a prevention target in mucosal abnormalities and hepatic steatosis. Midura-Kiela et al. [32] and Feng et al. [30] have reported that curcumin acts as an interferon gamma (IFN-γ) signaling inhibitor in colonocytes.

### 1.6. Curcumin and Microbiota in Colitis

Two independent studies [33,34] have explored the modulative effects of nanoparticle curcumin administration on colonic microbiota during colitis, and suggested a modulating structure of intestine biodiversity. Interestingly, the results of randomized, double-blind, placebo-controlled study published in 2021 showed that 8 weeks administration of Curcugen™, a curcumin extract (DolCas Biotech, LLC, 9 Lenel Rd, Landing, NJ 07850, USA), was correlated with an improvement in gastrointestinal symptoms (abdominal pain, indigestion, diarrhea, and constipation) in adults [35]. The curcumin extract Curcugen™ contains 98.5% turmeric-based ingredients (50% curcuminoids, 1.5% essential oils, and other native turmeric molecules). Additionally, the potential mechanisms associated with curcumin’s influence on microbial diversity have been examined. In comparison to placebo, no significant changes in the biodiversity (from the phylum to genus level) of bacteria which are regularly observed in adults with irritable bowel syndrome (IBS) were reported. These results could suggest that changes in the gut microbiota are not responsible for improving gastrointestinal symptoms in adults. In another previous study by McFadden et al., curcumin elevated microbial richness, prevented alpha diversity decrease, and increased the proportion of *Lactobacillales*, while conversely decreasing the proportion of *Coriobacterales* in mice, during colitis and colon cancer prevention [34].

### 1.7. Curcumin and Microbiota and Urinary Metabolism

Another interesting in vivo study has been carried out in healthy adults [36]. The aim of this study was to describe the potential effects of curcumin on changes in 24-hour urinary composition in healthy volunteers in response to daily consumption of a dried *Curcuma longa* extract containing a standardized amount of curcuminoid (equivalent to 100 mg of curcuminoids) for 28 days. Their results showed curcumin-induced alterations in urinary metabolites. Curcumin and two metabolic derivates (HTC and DHC) were detected in the urine, this fact points to the absorption of the major curcuminoid from the extract and its further metabolism by the liver and intestinal microflora.

Peterson et al. [5], in their pilot study, examined gut microflora profiles in healthy humans. All study participants were blinded to the treatments they received (curcumin tablets, turmeric, or placebo). The results concluded that the respondent’s microbiota response was individualized over time; however, the comparison of microbiota alterations in the turmeric and curcumin supplementation groups were highly similar. The authors interpreted this observation of the turmeric response as a reflection of the catabolism of polysaccharide compounds in the root, involving a wide range of glycosyl hydrolases encoded by the species, which were elevated in the responsive subjects (*Bacteroides, Bifidobacterium, Alistipes, Parabacteroides*).

### 1.8. Curcumin and Microbiota in Exercise Performance

There is some evidence suggesting that exercise training has an effect on gut microbiota, through its impact on the autonomic nervous system (vagal tone) by improving gut microbiota composition [37]. Chen et al. [38] investigated the effect of nano-bubble curcumin extract (NCE-5x) supplementation on microbiota and exercise performance in mice. They found that six weeks of nano-bubble curcumin extract treatment have modulated the gut microbiota composition and led to anti-fatigue effects, which resulted in improved exercise performance in the mice. Animals following the nano-curcumin extract had a reduced amount of *Bacteroidetes* and an elevated amount of *Firmicutes.* The number of bacteria from the family *Lactobacillaceae* and *Lactobacillus* genus was higher in the NCE-5x group, in comparison to the vehicle group. However, the amount of *Clostridiales* and *Allobaculum* were lower in the NCE-5x group than in the vehicle group. Therefore, there seems to be a definite need for establishing an optimal dosage of curcumin which is safe and effective, as a result of further studies in this field, especially in humans.

### 1.9. Curcumin and Microbiota in Dental Disease

Interestingly, some studies have shown the effects of curcumin on oral bacteria associated with dental disease. Li et al. [39] evaluated the inhibitory properties of curcumin on the metabolism and the formation of a biofilm in planktonic *Streptococcus mutans*, which has been shown to be a main etiological factor in dental caries [40]. They exhibited its therapeutic antibacterial activity by proving that curcumin decreased the biofilm metabolism after both 5 min and 24 h, thus inhibiting the number of live and total bacteria in the short- and long-term. The in vitro biofilm models of *S. mutans* were treated with different concentrations of curcumin. To analyze the composition and extracellular polysaccharide content of *S. mutans* biofilm after curcumin treatment, the researchers used confocal laser scanning microscopy. 

Moreover, their study revealed that curcumin treatment decreased not only the two-component transduction system but also metabolism adherence, carbohydrate metabolism, and the expression of genes that are linked to the synthesis of extracellular polysaccharide. Curcumin did not demonstrate any effect on the growth kinetics of *S. mutans*, so the reduced number of alive microorganisms in the study indicated a direct effect of curcumin on *S. mutans*. Despite the fact that Li et al. have explored the antibacterial effect of curcumin on *S. mutans*, it is important to take into account that the bacterial strains of *S. mutans* are heterogenic, with a variety of strains. Therefore, many more strains of *S. mutans* in the future should be assessed, in order to obtain more relevant results [39]. Table 1 lists some of the alterations in the number of gut microbes after curcumin treatment.

### 1.10. Curcumin and Metabolic Health

Numerous studies have shown that the changes in the microbiome are associated with many metabolic conditions which include among others obesity, diabetes, and chronic liver disease. These entities may be linked with changes in intestinal microbiome [28]. The first study reporting on the association between curcumin ingestion and the diversity of gut microbiome in a menopausal rat model was published in 2017 [41]. Their results suggested that curcumin may partially reverse the alterations in the biodiversity of rat gut microbiota by the changed distribution of intestinal microflora due to estrogen deficiency induced by ovariectomy. Curcumin promoted increases in the number of species of *Serratia, Shewanella, Pseudomonas, Papillibacter,* and *Exiguobacterium*, and decreases in *Anaerotruncus* and *Helicobacter pylori*. Moreover, Wang et al. [6] have delineated the underlying mechanism of the association of curcumin with metabolic diseases and chronic inflammation, through modulating the function of intestinal epithelial cells (IECs) and the intestinal barrier function. They used the human IEC lines immortalized cell line of human colorectal adenocarcinoma cells (Caco-2) and human colorectal adenocarcinoma cell line with epithelial morphology (HT-29), in this case examining the modulation effects of LPS on intracellular signaling as well as tight junctions. The presented results showed that curcumin attenuated the activation of IECs as well as macrophages and as a consequence led to decreased secretion of interleukin 1β (IL-1β). It is worth mentioning that IECs are activated by luminal LPS, which stimulates (through intestinal macrophages) proinflammatory cytokines. Circulating LPS levels and metabolic diseases (e.g., type 2 diabetes and atherosclerosis) have been associated. Western-type diets rich in high-fat foods induce alterations in the gut microbiota by releasing bacteria-derived products (e.g., LPS). Zam W. [42], in his review, mentioned that treatment with the nanoparticle curcumin (named Theracurmin) reduced mucosal mRNA expression of inflammatory mediators and the activation of nuclear factor kappa-light-chain-enhancer of activated B cells (NF-κB) in colonic epithelial cells. This has resulted in the elevation of the amount of butyrate-producing bacteria and fecal butyrate levels. The results of this study have been examined in experimental colitis mice [33].

Kato et al. [43] used curcumin dispersed in colloidal nanoparticles and stimulated the secretion of glucagon-like peptide 1 (GLP-1) thus leading to an increased synthesis and secretion of insulin, which resulted in a better glycemia control. Oral administration of highly dispersible and bioavailable curcumin, through the stimulation of insulin secretion in vivo, resulted in lower glycemia. Curcumin was administrated prior to IP glucose injection to confirm that the hypoglycemic and insulin secretion effects were linked to the stimulation of GLP-1 secretion. This finding suggests a potential role of curcumin in the treatment of diabetes mellitus. However, the use of native curcumin did not achieve therapeutic outcomes and did not improve glucose tolerance in mice.

Another interesting study has determined the effects of different nano-curcumin levels, and have revealed beneficial effects on growth, cholesterol levels, blood constitution, antioxidant indices, immunity, and growth of quails, as well as increased amounts of lactic acid bacteria and reduced amounts of pathogenic bacteria. In the study conducted by Reda et al. [44], liver function reflected by the alanine aminotransferase (ALT) and asparate aminotransferase (AST) levels significantly improved following the addition of dietary nano-curcumin. The addition of any amount of nano-curcumin in the quail diet also significantly improved the lipid profile. Moreover, the caecal microbiota of quail was altered, following the supplementation of nano-curcumin levels. Reductions in *Enterobacter*, *Salmonella*, and *E. coli* counts were observed. However, they highlighted the use of nanoparticles, such as nanocurcumin, which more easily pass through cell membranes and interplay promptly with biological systems.

## 2. Summary

Polyphenols, such as curcuminoids, are naturally occurring bioactive compounds that, due to their antioxidant abilities, play important roles in human nutrition. A substantial amount of promising evidence has indicated that curcumin may be capable of preventing and combating several metabolic syndromes, cancer, and obesity, and may even play a neuroprotective role. The metabolism of curcumin, which occurs in the intestine, enhances its biological activity and, as a consequence, biotransforms it into active metabolites, which may promote beneficial effects in the gut microbiota. However, there are also many limitations, such as its poor bioavailability, determining the dosage level of curcumin to achieve the optimal health results, and limitations related to animal studies. Currently, based on available data, there are several contraindications for use of cur-cumin. Curcumin should be used with caution given the dual effects of curcumin on alcoholic liver injury in people with liver disease, like cirrhosis, biliary obstruction, gallstones, biliary colic, and people abusing alcohol. In addition, curcumin may interact with nonsteroidal anti-inflammatory drugs, reserpine, and blood thinners. Therefore, the use of curcumin in people who are taking those drugs is also not recommended. Another important limitation is that curcumin cannot be tested in randomized placebo-controlled studies. Hence, there are much more difficulties to conduct research in any given clinical condition. Moreover, curcumin metabolism may be different from person to person due to the individual’s variety of microorganism content. At present, extrapolation of animal studies has lead to the recommendation of administering oral curcumin in the amount of about 500 mg per day (about 4 g raw curcumin). The use of nano-curcumin is one of the possible mechanism for enhancing the bioavailability of curcumin increasing its absorption. Nanoparticles such as nanocurcumin seem to give the best effects on gut microbiota and have shown the best properties for the body.

In summary, many studies have indicated that a curcumin intervention using a high-bioavailability formulation would be more effective than treatment with standard curcumin, even considering a longer period of time. Additionally, some considerations for the effective dosage might be specific to the particular formulation in question, as well as the bioavailability of the curcumin within the compound. Future studies should focus on providing more comprehensive data concerning high-bioavailability curcumin supplementation, especially in humans. There is also a need to better understand the bi-directional metabolism of curcumin, as well as its potential beneficial effects on microbiota and overall health.

## Figures and Tables

**Table 1:** Alteration in gut microbiota after curcumin treatment.

Dose of Curcumin	Alterations in Gut Microbiota	*p*	Author, Year, [Reference]
100 mg/kg body weight of mice (15 days)	Decrease: *Prevotellaceae* (15.48% -> 6.16%) *Prevotella* (13.29% -> 4.63%) Increase: *Bacteroidaceae* (3.21% -> 1.15%) *Rikenellaceae* (4.73% -> 7.96%) *Alistipes* (4.73% -> 7.96%) *Bacteroides* (1.15% -> 3.21%)	*p* = 0.01 *p* = 0.00 *p* = 0.00 *p* = 0.04 *p* = 0.04 *p* = 0.00	Shen L., 2017 [28]
200 mg/kg body weight of rats (4 weeks) after HFD (high-fat diet)	Decrease: *Spirochaetae* (0.0091%) *Tenericutes* (0.013%) *Elusimicrobia* (0.0045%) Increase: *Actinobateria* (7.47%) *Collinsella* (7.18%) *Streptococcus* (0.66%) *Suterella* (0.23%) *Gemella* (0.09%) *Thalassospira* (0.26%) *Gordonibacter* (0.071%) *Actinomyces* (0.038%)	*p* < 0.05	Feng W., 2017 [30]
8–162 mg/kg body weight of mice during colitis and colon cancer prevention (the entire study lasted 30 weeks)	Decrease: *Coriobacterales* Increase: *Lactobacillales*		McFadden R.M.T., 2015 [34]
Pilot study in humans (three groups: placebo, turmeric, and curcumin) tablets 1000 mg of curcumin + 1.25 mg black pepper)	Decrease: *Blautia* spp. Increase: *Clostridium* spp. *Bacteroides* spp. *Citrobacter* spp. *Cronobacter* spp. *Enterobacter* spp. *Enterococcus* spp. *Klebsiella* spp. *Parabacteroides* spp. *Pseudosomonas* spp.		Peterson C.T., 2018 [5]
100 mg/kg body weight of rats (three groups: ovariectomized, sham operation, curcumin)	Decrease: *Anaerotruncus* *Helicobacter* Increase: *Serratia* *Shewanella* *Pseudomonas* *Papillibacter* *Exiguobacterium*	*p* = 0.004 *p* = 0.049 *p* = 0.002 *p* = 0.006 *p* = 0.014 *p* = 0.029 *p* = 0.032	Zhang Z., 2017 [41]
Male mice divided into 3 groups: Vehicle, 0; NCE-1x, 3.075 g/kg^-1^ day^-1^; NCE-5x, 15.375 g/kg^-1^ day^-1^	Increased: *Firmicutes* *Lactobacillaceae* *Lactobacillus* Decreased: *Bacteroidetes* *Clostridiales* *Allobaculum*	*p* < 0.05	Chen Y-M., 2020 [38]

Abbreviations: HFD—high fat diet; NCE-1x—nano-bubble curcumin extract-1x; NCE-5x—nano-bubble curcumin extract-5x.

## References

[1] Shen L., Ji H.F. (2019). Bidirectional interactions between dietary curcumin and gut microbiota. Crit. Rev. Food Sci. Nutr..

[2] Shabbir U., Rubab M., Daliri E.B.-M., Chelliah R., Javed A., Oh D.H. (2021). Settings Open Access Review Curcumin, Quercetin, Catechins and Metabolic Diseases: The Role of Gut Microbiota. Nutrients.

[3] Nisari M., Yilmaz S., Ertekin T., Hanim A.Y., Ceylan D., Inanc N., Al Ö., Ülger H. (2020). Effects of curcumin on lipid peroxidation and antioxidant enzymes in kidney, liver, brain and testis of mice bearing Ehrlich Solid Tumor. Adv. Hyg. Exp. Med..

[4] Shabbir U., Rubab M., Tyagi A., Oh D.H. (2021). Curcumin and Its Derivatives as Theranostic Agents in Alzheimer’s Disease: The Implication of Nanotechnology. Int. J. Mol. Sci..

[5] Peterson C.T., Vaughn A.R., Sharma V., Chopra D., Mills P.J., Peterson S.N., Sivamani R.K. (2018). Effects of Turmeric and Curcumin Dietary Supplementation on Human Gut Microbiota: A Double-Blind, Randomized, Placebo-Controlled Pilot Study. J. Evid. Based Integr. Med..

[6] Wang J., Siddhartha S., Ghosh S. (2017). Curcumin improves intestinal barrier function: Modulation of intracellular signaling, and organization of tight junctions. Am. J. Physiol. Cell Physiol..

[7] Adiwidjaja J., McLachlan A.J., Boddy A.V. (2017). Curcumin as a clinically-promising anti-cancer agent: Pharmacokinetics and drug interactions. Expert Opin. Drug Metab. Toxicol..

[8] Sharma R.A., Euden S.A., Platton S.L., Cooke D.N., Shafayat A., Hewitt H.R., Marczylo T.H., Morgan B., Hemingway D., Plummer S.M. (2004). Phase I clinical trial of oral curcumin: Biomarkers of systemic activity and compliance. Clin. Cancer Res..

[9] Di Meo F., Margarucci S., Galderisi U., Crispi S., Peluso G. (2019). Curcumin, Gut Microbiota, and Neuroprotection. Nutrients.

[10] Dei Cas M., Ghidoni R. (2019). Dietary Curcumin: Correlation between Bioavailability and Health Potential. Nutrients.

[11] Jardim K.V., Neves Siqueira J.L., Bao S.N., Sousa M.H., Parize A.L. (2020). The role of the lecithin addition in the properties and cytotoxic activity of chitosan and chondroitin sulfate nanoparticles containing curcumin. Carbohydr. Polym..

[12] Maryam S.H.-Z., Sarhadi M., Zarei M., Thilagavathi R., Selvam C. (2021). Synergistic effects of curcumin and its analogs with other bioactive compounds: A comprehensive review. Eur. J. Med. Chem..

[13] Hewlings S.J., Kalman D.S. (2017). Curcumin: A Review of Its Effects on Human Health. Foods.

[14] Schiborr C., Kocher A., Behnam D., Jandasek J., Toelstede S., Frank J. (2014). The oral bioavailability of curcumin from micronized powder and liquid micelles is significantly increased in healthy humans and differs between sexes. Mol. Nutr. Food Res..

[15] Scazzocchio B., Minghetti L., D’Archivio M. (2020). Interaction between Gut Microbiota and Curcumin: A New Key of Understanding for the Health Effects of Curcumin. Nutrients.

[16] Ng Q.X., Sof A.Y.S., Loke W., Venkatanarayanan N., Lim D.Y., Yeo W.S. (2018). A Meta-Analysis of the Clinical Use of Curcumin for Irritable Bowel Syndrome (IBS). J. Clin. Med..

[17] Pandey A., Chaturvedi M., Mishra S., Kumar P., Somvanshi P., Chaturvedi R. (2020). Reductive metabolites of curcumin and their therapeutic effects. Heliyon.

[18] Paulraj F., Abas F., Lajis N.H., Othman I., Naidu R. (2019). Molecular Pathways Modulated by Curcumin Analogue, Diarylpentanoids in Cancer. Biomolecules.

[19] Ireson C.R., Jones D.J.L., Orr S., Coughtrie M.W.H., Boocock D.J., Williams M.L., Farmer P.B., Steward W.P., Gescher A.J. (2002). Metabolism of the cancer chemopreventive agent curcumin in human and rat intestine. Cancer Epidemiol. Biomark. Prev..

[20] Hassaninasab A., Hashimoto Y., Tomita-Ypokotani K., Koboyashi M. (2011). Discovery of the curcumin metabolic pathway involving a unique enzyme in an intestinal microorganism. Proc. Natl. Acad. Sci. USA.

[21] Hoehle S.I., Pfeiffer E., Solyom A.M., Metzler M. (2006). Metabolism of Curcuminoids in Tissue Slices and Subcellular Fractions from Rat Liver. J. Agric. Food Chem..

[22] Xiao-Yu X., Xiao M., Li S., Ren-You G., Ya L., Hua-Bin L. (2018). Bioactivity, Health Benefits, and Related Molecular Mechanisms of Curcumin: Current Progress, Challenges, and Perspectives. Nutrients.

[23] Pluta R., Januszewski S., Ułamek-Kozioł M. (2020). Mutual Two-Way Interactions of Curcumin and Gut Microbiota. Int. J. Mol. Sci..

[24] Burapan S., Kim M., Han J. (2017). Curcuminoid Demethylation as an Alternative Metabolism by Human Intestinal Microbiota. J. Agric. Food Chem..

[25] Wang J., Yu X., Zhang L., Wang L., Peng Z., Chen Y. (2018). The pharmacokinetics and tissue distribution of curcumin and its metabolites in mice. Biomed. Chromotography.

[26] Sun Z.Z., Li X.Y., Wang S., Shen L., Ji H.F. (2020). Bidirectional interactions between curcumin and gut microbiota in transgenic mice with Alzheimer’s disease. Appl. Microbiol. Biotechnol..

[27] Jazayeri S.D. (2009). Survival of Bifidobacteria and other selected intestinal bacteria in TPY medium supplemented with Curcumin as assessed in vitro. Int. J. Probiotics Prebiorics.

[28] Shen L., Liu L., Hong-Fang J. (2017). Regulative effects of curcumin spice administration on gut microbiota and its pharmacological implications. Food Nutr. Res..

[29] Bereswill S., Munoz M., Fisher A., Plickert R., Haag L.M., Otto B., Kuhl A.A., Loddenkemper C., Gobel U.B., Heimesaat M.M. (2010). Anti-Inflammatory Effects of Resveratrol, Curcumin and Simvastatin in Acute Small Intestinal Inflammation. PLoS ONE.

[30] Feng W., Wang H., Zhang P., Gao C., Tao J., Ge Z., Zhu D., Bi Y. (2017). Modulation of gut microbiota contributes to curcumin-mediated attenuation of hepatic steatosis in rats. Biochim. Biophys. Acta.

[31] Zhao Y., Zhang M., Pang X., Xu J., Kang C., Li M., Zhang C., Zhang Z., Zhang Y., Li X. (2012). Structural Changes of Gut Microbiota during Berberine-Mediated Prevention of Obesity and Insulin Resistance in High-Fat Diet-Fed Rats. PLoS ONE.

[32] Midura-Kiela M.T., Radhakrishnan V.M., Marmonier C.B., Laubtiz D., Ghishan F.K., Kiela P.R. (2012). Curcumin inhibits interferon-γ signaling in colonic epithelial cells. Am. J. Physiol. Gastrointest. Liver Physiol..

[33] Ohno M., Nishidia A., Sugitani Y., Nishino K., Inatomi O., Sugimoto M., Kawahara M., Andoh A. (2017). Nanoparticle curcumin ameliorates experimental colitis via modulation of gut microbiota and induction of regulatory T cells. PLoS ONE.

[34] McFadden R.M.T., Larmonier C.B., Shehab K.W., Midura-Kiela M., Ramalingam R., Harrison C.A., Besselsen D.G., Chase J.H., Caporaso J.G., Jobin C. (2015). The Role of Curcumin in Modulating Colonic Microbiota during Colitis and Colon Cancer Prevention. Inflamm. Bowel Dis..

[35] Lopresti A.L., Smith S.J., Rea A., Michel S. (2021). Efficacy of a curcumin extract (Curcugen™) on gastrointestinal symptoms and intestinal microbiota in adults with self-reported digestive complaints: A randomized, double-blind, placebo-controlled study. BMC Complementary Med. Ther..

[36] Peron G., Sut S., Dal Ben S., Voinovich D., Dall’Acqua S. (2020). Untargeted UPLC-MS metabolomics reveals multiple changes of urine composition in healthy adult volunteers after consumption of curcuma longa L. extract. Food Res. Int..

[37] Campbell M.S., Carlini N.A., Fleenor B.S. (2020). Infulence of curcumin on performance and post-exercise recovery. Crit. Rev. Food Sci. Nutr..

[38] Chen Y.-M., Chiu W.-C., Chiu Y.-S., Li T., Sung H.-S., Hsiao C.-Y. (2020). Supplementation of nano-bubble curcumin extract improves gut microbiota composition and exercise performance in mice. Food Funct..

[39] Li B., Li X., Lin H., Zhou Y. (2018). Curcumin as a Promising Antibacterial Agent: Effects on Metabolism and Biofilm Formation in S. mutans. BioMed Res. Int..

[40] Bowen W.H., Koo H. (2011). Biology of Streptococcus mutans-Derived Glucosyltransferases: Role in Extracellular Matrix Formation of Cariogenic Biofilms. Caries Res..

[41] Zhang Z., Chen Y., Xiang L., Wang Z., Xiao G.G., Hu J. (2017). Effect of Curcumin on the Diversity of Gut Microbiota in Ovearectomized Rats. Nutrients.

[42] Zam W. (2018). Gut Microbiota as a Prospective Therapeutic Target for Curcumin: A Review of Mutual Influence. J. Nutr. Metab..

[43] Kato M., Nishikawa S., Ikehata A., Dochi K., Tani T., Takahashi T., Imaizumi A., Tsuda T. (2016). Curcumin improves glucose tolerance via stimulation of glucagon-like peptide-1 secretion. Mol. Nutr. Food Res..

[44] Reda F.M., El-Saadony M.T., Elnesr S.S., Alagawany M., Tufarelli V. (2020). Effect of Dietary Supplementation of Biological Curcumin Nanoparticles on Grwoth and Carcass Traits, Antioxidant Status, Immunity and Caecal Microbiota of Japanese Quails. Animals.

